# Role of cardiac ^123^I-*m*IBG imaging in predicting arrhythmic events in stable chronic heart failure patients with an ICD

**DOI:** 10.1007/s12350-018-1258-z

**Published:** 2018-03-28

**Authors:** Giuseppe De Vincentis, Viviana Frantellizzi, Francesco Fedele, Alessio Farcomeni, Paola Scarparo, Nicolò Salvi, Danilo Alunni Fegatelli, Massimo Mancone, Derk O. Verschure, Hein J. Verberne

**Affiliations:** 1 Department of Radiological Sciences, Oncology and Anatomo-Pathology, Sapienza - University of Rome, Rome, Italy; 2 Angio-Cardio-Thoracic Pathophysiology and Imaging, Sapienza - University of Rome, Rome, Italy; 3 Department of Cardiovascular, Respiratory, Nephrology, Anesthesiology and Geriatric Sciences, Sapienza - University of Rome, Rome, Italy; 4 Department of Public Health and Infectious Diseases, Sapienza - University of Rome, Rome, Italy; 5 Department of Cardiology, Zaans Medical Center, Zaandam, The Netherlands; 6 Department of Radiology and Nuclear Medicine, Academic Medical Center, University of Amsterdam, Meibergdreef 9, 1105 AZ Amsterdam, The Netherlands

**Keywords:** ^123^I-*m*IBG scintigraphy, ICD, chronic heart failure, planar, SPECT, heart-to-mediastinum ratio, washout

## Abstract

**Background:**

Despite therapeutic improvement, the prognosis of chronic heart failure (CHF) remains unfavorable partly due to arrhythmia and sudden cardiac death (SCD). This prospective study evaluated myocardial ^123^I-*meta*-iodobenzylguanidine (^123^I-*m*IBG) scintigraphy as a predictor of arrhythmic events (AE) in CHF patients.

**Methods:**

170 CHF patients referred for implantable cardioverter-defibrillator (ICD) implantation for both primary and secondary prevention were enrolled. All patients underwent planar and SPECT imaging. Early and late heart-to-mediastinum (H/M) ratio, ^123^I-*m*IBG washout (WO), early and late summed SPECT scores were calculated The primary endpoint was an AE: sustained ventricular tachycardia, resuscitated cardiac arrest, appropriate ICD therapy or SCD. The secondary endpoint was appropriate ICD therapy.

**Results:**

During a median follow-up of 23.3 months, 69 patients experienced an AE. Early summed score (ESS) was the only independent predictor of AE [HR 1.023 (1.003-1.043)]. Focussing on only patients with an ICD for primary prevention, ESS was the only independent predictor of AE [HR 1.028 (1.007-1.050)]. ^123^I-*m*IBG-derived parameters failed to be independent predictors of appropriate ICD therapy. However there was a “bell-shaped” relation between ^123^I-*m*IBG scintigraphy-derived parameters and AE and appropriate ICD therapy, i.e., those with intermediate ^123^I-*m*IBG abnormalities tended to be at higher risk of events.

**Conclusion:**

Although SPECT ^123^I-*m*IBG scintigraphy was associated with AE in CHF patients with ICD implantation for primary and secondary prevention, no association was found between ^123^I-*m*IBG scintigraphy-derived parameters and appropriate ICD therapy.

**Electronic supplementary material:**

The online version of this article (10.1007/s12350-018-1258-z) contains supplementary material, which is available to authorized users.

## Introduction

Chronic heart failure (CHF) is a life-threatening syndrome with a growing incidence and prevalence over the last decades, with consequently high hospitalization and re-hospitalization rates. CHF is associated with high morbidity and mortality rates and approximately 50% of deaths are related to sudden cardiac death (SCD). In addition, mortality rates increase with increased disease duration, i.e., 20% during the first year and rising to approximately 50% within 5 years.^1^

In order to prevent SCD in CHF patients, current guidelines recommend an implantable cardioverter-defibrillator (ICD) in those patients with a left ventricular ejection fraction (LVEF) < 35%, NYHA class II or III and treated with optimal medical therapy. Initial trials showed that ICD’s are effective in reducing SCD in CHF patients.^2^ However, recent studies suggest that several ICD recipients experience inappropriate shocks and ICD-associated infectious complications.^3^–^5^ Furthermore, some arrhythmic deaths happen in patients who were classified as low risk for arrhythmic cardiac death and had no ICD indication. In addition, for example, in the SCD-HeFT (Sudden Cardiac Death in Heart Failure Trial) study three years after ICD implantation for primary prevention, a remarkably high percentage (65%) of patients had never received appropriate ICD therapy.^6^ Taken together, these issues suggest that the current ICD selection criteria in CHF patients, now largely driven by LVEF, are suboptimal.

The cardiac sympathetic activity is one of the neurohormonal compensation mechanisms that plays an important role in the pathogenesis and prognosis of CHF with impaired LVEF. Initially, beta-adrenergic receptor stimulation by increased synaptic norepinephrine (NE) levels helps to compensate for impaired myocardial function, but long-term NE excess has detrimental effects on myocardial structure and gives rise to a downregulation and decrease in the sensitivity of post-synaptic β-adrenergic receptor and consequently, poor outcome.^7^

Cardiac sympathetic activity can non-invasively be assessed with *meta*-iodobenzylguanidine (^123^I-*m*IBG) using both planar and SPECT techniques.^8^^123^I-*m*IBG is a radiolabeled NE analogue and accumulates in the presynaptic myocardial sympathetic nerve endings. Quantified myocardial ^123^I-*m*IBG parameters have proved to be of prognostic value in CHF.^9^–^12^ Those patients with impaired myocardial ^123^I-*m*IBG parameters had a worse prognosis compared with those with relatively preserved parameters (i.e., reduced late heart-to-mediastinum (H/M) ratio and increased ^123^I-*m*IBG myocardial washout (WO)).

More specifically, Boogers et al. demonstrated that high values of the SPECT-derived summed myocardial denervation score, (i.e., extensively reduced activity of the norepinephrine transporter was associated with an increased risk of ventricular arrhythmia related to appropriate ICD therapy and SCD.^10^ However, the study population consisted of a heterogeneous failure population with an ICD for both primary and secondary prevention.

The primary goal of our study was to evaluate the efficacy of both planar and SPECT ^123^I-*m*IBG imaging to predict AE, defined as an episode of sustained ventricular tachycardia, resuscitated cardiac arrest, appropriate ICD discharge (anti-tachycardial pacing or defibrillation), or SCD. In addition, we studied whether ^123^I-*m*IBG scintigraphy could predict appropriate ICD therapy specifically (i.e., secondary endpoint).

## Materials and Methods

We performed a prospective, single-center observational study of CHF patients with an indication for ICD implantation as for both primary and secondary prevention. From October 2011 to January 2016, consecutive CHF patients, fulfilling the inclusion criteria, were prospectively enrolled.

Inclusion criteria were NYHA class II or III; LVEF < 35%; indication for an ICD implantation (primary or secondary prevention); expected survival > 1 years; age > 18 years old; signed informed consent. All patients received optimal medical therapy according to current guidelines.^13^ Exclusion criteria were previous ICD implantation; cardiac resynchronisation therapy (CRT) indication; cancer history; severe valvulopathy; recent acute coronary syndrome (< 3 months); contraindication to ICD implantation.

Enrolled patients underwent ^123^I-*m*IBG scintigraphy 7-15 days before ICD implantation. Settings for detection of ventricular tachycardia’s or fibrillation were at the discretion of the implanting physician.

Clinical follow-up with ICD interrogation was performed at 6, 12, and 18 months. AE were defined as episodes of sustained ventricular tachycardia (> 30 seconds), resuscitated cardiac arrest, appropriate ICD intervention (anti-tachycardial pacing or defibrillation), or SCD.

Written informed consent was obtained from all patients. The study protocol was approved by the institutional committee on human research and conducted in accordance with the ethical guidelines of the 1975 Helsinki Declaration.

### Image Acquisition of Planar and SPECT ^123^I-*m*IBG Scintigraphy

A 5% Lugol solution was administered to block the thyroid before the administration of 150-185 MBq of ^123^I-*m*IBG (AdreView, GE Healthcare), resulting in an effective dose of 2.5 to 3.3 mSv.

Fifteen (i.e., early) and 240 (i.e., late) minutes after tracer injection, planar anterior thoracic images were acquired for 10 minutes with a zoom factor of 1 and stored in a 128 × 128 matrix. The images were acquired in supine position, with a dual-head gamma camera (Infinia, GE Healthcare, Milwaukee, USA) using a low energy parallel-hole high-resolution collimator (LEHR).

Immediately after the planar images, at 25 minutes and 250 minutes after injection, SPECT cardiac images were acquired with the dual-headed gamma camera over 180° using a 90°-rotation, starting at 45° right-anterior oblique projection and proceeding to the 45° left-posterior oblique projection. A 64 × 64 matrix was used for the SPECT studies (zoom factor 1). We applied a step-and-shot technique (64 projections, 30 seconds of duration per frame in non-gated mode). For both planar and SPECT, the energy window was symmetrically centered to ± 10% of the 159-KeV ^123^I photopeak.

### Planar ^123^I-*m*IBG Scintigraphy Analysis

Planar ^123^I-*m*IBG images were analyzed to obtain semi-quantitative parameters of tracer distribution. On the planar images, a manually polygonal region of interest (ROI) was drawn over the heart, around the epicardial border and the valve plane, including the left ventricular cavity (H), being careful to exclude lung and liver from the myocardial ROI.^14^ A second 7 × 7 pixel square ROI was placed on the upper half of the mediastinum (M), below the thyroid gland. H/M ratio’s, at 15 minutes (early H/M ratio) and 240 minutes (late H/M ratio), were calculated as mean counts per pixel in the myocardial ROI divided by the mean counts per pixel in the mediastinal ROI.^14^

The myocardial washout rate (WO) from early to late images was calculated using background correction according to the following formula and expressed as a percentage:^14^

$$ {\text{WO }} = \frac{{\left\{ {\left[ {{\text{He}} - {\text{Me}}} \right]  {-} \left[ {\left( {{\text{Hl}} - {\text{Ml}}} \right) \times 1.21} \right]} \right\}}}{{\left[ {{\text{He}} - {\text{Me}}} \right]}} \times 100 $$

where 1.21 is the correction for ^123^I decay at 3 hours and 45 minutes; e the early images; l the late images; H the heart counts per pixel; and M is the mediastinal counts per pixel.

### SPECT ^123^I-*m*IBG Scintigraphy Analysis

**Figure 1:**
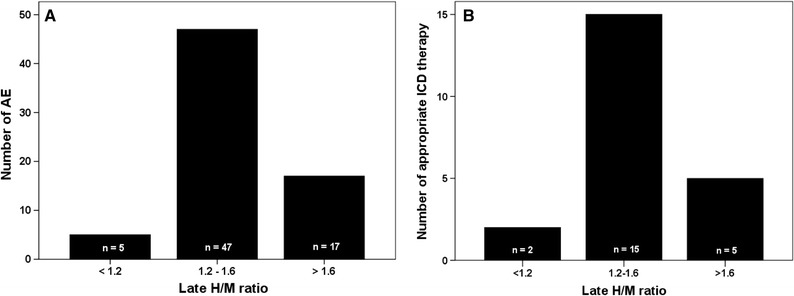
(**A**) Relation between late H/M ratio categories and arrhythmic events (AE). (**B**) Relation between late H/M ratio categories and appropriate ICD therapy. Due to a relatively low number of patients with late H/M ratio < 1.2 (*n* = 8) compared to those with late H/M ratio > 1.6 (*n* = 51) and intermediate late H/M ratio (*n* = 111), no adequate statistical analysis was feasible to compare the three groups. Therefore, we combined the groups with late H/M ratio < 1.2 and > 1.6 and compared this combined group with those with an intermediate late H/M ratio (1.2–1.6): *P* = .014 for AE and *P* < .001 for ICD therapy

Using Myovaton software implemented on a Xeleris Duo platform (GE Healthcare), SPECT images were reoriented in short, vertical long, and horizontal long heart axes after filtered-back-projection (FBP) reconstruction. A low-pass filter, Butterworth (order: 10, cut-off frequency: 0.3 cycles/cm for early SPECT and order: 20, cut-off frequency: 0.3 cycles/cm for late SPECT) was used as a preprocessing filter; no scatter or attenuation correction was applied (Figure 1). After reorientation of myocardial axes, heart images were analyzed with a standard 17-segments model similarly to the conventional methods used for myocardial perfusion imaging (MPI).^15^,^16^ All 17 segments were visually scored by each reader using a 5-point scale where myocardial ^123^I-*m*IBG uptake was expressed as a percentage of the maximum myocardial uptake^17^: segments with a value greater than 70% were defined as normal and scored as 0. Segments with mildly reduced uptake, ranging from 69 to 60%, were scored as 1. A score of 2 was assigned to segments with moderately reduced uptake, 59-50%. Segments with severely reduced uptake, 49-40%, were scored as 3. Finally, segments with absent uptake (i.e., lower than 39%) were scored as 4. The defect scores were calculated as the sum of the segmental scores and the early and late summed scores (ESS and LSS, respectively) were obtained. The difference summed score (DSS) was obtained as the subtraction between ESS and LSS.

### Observer Variability of SPECT ^123^I-*m*IBG Images

For the inter-observer analysis, three observers with different degrees of expertise in nuclear cardiology techniques, independently reviewed all SPECT ^123^I-*m*IBG images.

### Statistical Analysis

Data are expressed as means ± standard deviation, differences between groups (those with AE and those without AE) were evaluated with the independent samples T-test, the Mann-Whitney U test, or χ^2^ when appropriate. Incidence of events was estimated by means of Kaplan-Meier curves, and univariate or multivariable Cox regression models were used to assess (adjusted) hazard ratios. Proportionality of hazards was checked by means of residual analysis. Time-dependent ROC curves from censored data were computed using the Kaplan-Meier method.^18^,^19^ In order to assess reproducibility among three operators, the intra-class correlation coefficient (ICC) was used. For clinically relevant agreement, the following criteria were used: ICC values < .40, .40-.59, .60-.74, .75-1.00 were considered poor, fair, good, excellent, respectively.

A *P* value < .05 was considered to indicate a statistical significance. All analyses were conducted with R software version 3.0.2 (R Development Core Team, 2013).

## Results

### Subjects

A total of 170 consecutive patients (139 male, mean age 64.2 ± 12.6 years) were enrolled. The baseline characteristics are shown in Table 1. Almost 60% of the patients had ischemic heart disease. Eighty-six patients (50.6%) were classified as NYHA class II and 84 (49.4%) as NYHA class III. The mean LVEF was 31.1 ± 9.4%. Approximately 25% of the patients had a left bundle branch block (LBBB). The majority of patients (*n* = 156) received an ICD for primary prevention. The mean early H/M ratio was 1.62 ± .21, the late H/M ratio was 1.53 ± .23, and ^123^I-*m*IBG WO was 30.9 ± 18.4%. The mean early SPECT summed score (ESS) was 31.4 ± 11.9, the mean late SPECT score (LSS) was 36.2 ± 12.3, and the SPECT difference score (DSS) (i.e., ESS - LSS) was − 4.7 ± 8.6.

**Table 1:** Patient characteristics with subgroups arrhythmic event (AE) and no AE

	All (*n* = 170)	With AE (*n* = 69)	Without AE (*n* = 101)	*P* value
Sex, male (%)	139 (81.8)	47 (68.1)	92 (91)	.316
Age	64.2 ± 12.6	64.4 ± 13.1	64.2 ± 12.3	.942
BMI (kg/m^2^)	1.73 ± .59	1.77 ± .61	1.71 ± .48	.765
Diabetes (%)	61 (36.0)	25 (36.2)	36 (35.6)	.478
Hypertension (%)	134 (78.9)	54 (78.2)	80 (79.2)	.831
Smoker, current, or past (%)	65 (38.14)	23 (33.3)	42 (41.5)	.642
Dyslipidemia (%)	80 (47.2)	33 (47.8)	47 (46.5)	.566
Ischemic heart disease (%)	101 (59.4)	43 (62.3)	58 (57.4)	.526
LBBB (%)	43 (25.3)	21 (30.4)	22 (21.8)	.205
LVEF (%)	31.1 ± 9.4	30.9 ± 9.7	31.3 ± 9.4	.776
NYHA functional class (%)				
II	86 (50.6)	21 (30.5)	65 (64.4)	.189
III	84 (49.4)	48 (69.5)	36 (35.6)	
Medication				
ACE-I (%)	74 (43.5)	24 (34.8)	50 (49.5)	.081
ARB (%)	32 (18.8)	12 (17.4)	20 (19.8)	.845
MRA (%)	105 (61.7)	55 (79.7)	50 (49.5)	< .001
Beta-blocker (%)	162 (95.3)	69 (100.0)	93 (92.1)	.043
Amiodarone (%)	38 (22.3)	11 (15.9)	27 (26.7)	.141
Statin (%)	98 (57.7)	42 (60.9)	56 (55.4)	.586
Diuretic (%)	158 (93.0)	65 (94.2)	93 (92.1)	.821
^123^I-*m*IBG scintigraphy				
Early H/M ratio	1.62 ± .21	1.61 ± .21	1.64 ± .22	.416
Late H/M ratio	1.53 ± .23	1.49 ± .22	1.55 ± .24	.074
Washout	30.9 ± 18.4	34.8 ± 17.4	28.2 ± 18.7	.020
ESS	31.4 ± 11.9	34.2 ± 11.8	29.6 ± 11.6	.012
LSS	36.2 ± 12.3	38.5 ± 12.7	34.6 ± 11.8	.040
DSS	− 4.74 ± 8.6	− 4.35 ± 8.85	− 5.0 ± 8.4	.612

*P* values indicate difference between groups

*BMI*, body mass index; *NYHA*, New York Heart Association; *LVEF*, left ventricular ejection fraction; *LBBB*, left bundle branch block; *ACE-I*, angiotensin converting enzyme inhibitor; *ARB*, angiotensin II receptor blockers; *MRA*, mineral receptor blocker; *ESS*, early summed score; *LSS*, late summed score; *DSS*, difference summed score

### Arrhythmic Events

Follow-up was complete in all patients with a median follow-up of 23.3 (1-51) months. In total, 69 patients experienced an AE. Of these 25 had sustained ventricular tachycardia (> 30 seconds), 3 of whom had a spontaneous reconversion to sinus rhythm, 22 experienced an appropriate ICD therapy (anti-tachycardial pacing or defibrillation), 8 had a resuscitated cardiac arrest, and 14 died of SCD. Patients with AE tended to have a lower, but not significant late H/M ratio (*P* = .074), higher myocardial ^123^I-*m*IBG washout (*P* = .020) , and higher ESS and LSS (respectively, *P* = .012 and *P*= .040).

In the overall population (i.e., primary and secondary prevention), the late H/M ratio showed in relation to AE a “bell-shaped” curve (Figure 1A). A similar curve was found for late H/M ratio in relation to appropriate ICD therapy (Figure 1B). Patients with intermediate late H/M ratios (range 1.2-1.6) were more likely to have an AE and appropriate ICD therapy compared to patients with low and high late H/M ratios. A similar pattern was observed for the primary prevention group only (data not shown)

### SPECT ^123^I-*m*IBG Observer Variability

The ICC for inter-observer variability of the summed ^123^I*-m*IBG SPECT scores showed an excellent agreement for both ESS and LSS (.846, 95% CI: .801-.884, *P* < .001 and .829, 95% CI: .786-.866, *P* < .001, respectively).

### Independent Predictors of Arrhythmic Events

The results of the multivariate Cox regression analysis are shown in Table 2. ESS was the only independent predictor of AE in both primary and secondary prevention [HR 1.023 (1.003-1.043), *P* = .023]. Focussing on only patients with an ICD for primary prevention, the ESS was the only independent predictor of the combined AE [HR 1.028 (1.007-1.050), *P* = .009]. Due to the relative low number of patients with a secondary ICD indication, no subgroup analysis was performed for this subgroup.

**Table 2:** Multivariate Cox regression analysis for different cardiac events in the total study population and in the primary prevention population only

	Variable	HR (95%)	*χ*^2^	Change *χ*^2^	*P* value
Primary and secondary (*n* = 170)					
AE	ESS	1.023 (1.003–1.043)	5.221	5.154	.023
Appropriate ICD therapy	Late H/M ratio	.153 (.020–1.172)	3.238	3.580	.071
SCD	ESS	1.048 (1.000–1.098)	9.175	6.641	.050
	LVEF	.924 (.855–.998)		11.136	.045
Primary (*n* = 156)					
AE	ESS	1.028 (1.007–1.050)	6.998	6.929	.009
SCD	ESS	1.047 (.997–1.099)	8.753	6.175	.040
	LVEF	.924 (.855–.998)		10.866	.064

*AE*, arrhythmic events; *ICD*, implantable cardiac defibrillator; *SCD*, sudden cardiac death; *ESS*, early summed score; *LVEF*, left ventricular ejection fraction

When we analyzed the separate AE classifications in the combined group of primary and secondary ICD indication, age was the only independent predictor appropriate ICD therapy [HR .971 (.944-.999), *P* = .040]. For combined primary and secondary prevention, late H/M ratio failed to be an independent predictor of appropriate ICD therapy [HR .153 (.020-1.172), *P* = .071]. In addition, the late H/M ratio also failed as an independent predictor of appropriate ICD therapy in the primary prevention group only. Both the ESS and the LVEF were independent predictors of SCD [respectively, HR 1.048 (1.000-1.098), *P* = .050 and HR .924 (.855-.998), *P* = .045].

## Discussion

CHF is associated with high morbidity and mortality rates. Despite optimal medical therapy, arrhythmia and SCD occur frequently in this population. In the past decades, ICD implantation has become an integral component of CHF management.^13^ Although the efficacy of ICD implantation has been proven in many multicenter studies, questions have been raised if patients selection is adequate. Even though LVEF < 35% is most commonly used for risk stratification for ventricular arrhythmias, it does not adequately identify patients at risk for SCD. In the MADIT II population, only 35% of the patients received appropriate ICD therapy during 3-year follow-up.^6^

The present study demonstrates that in stable CHF patients with ICD implantation for both primary and secondary prevention, ESS was the only SPECT ^123^I-*m*IBG scintigraphy-derived parameter associated with AE. The planar-derived parameters (i.e., early and late H/M ratios and ^123^I-*m*IBG WO) were not able to predict AE. Furthermore, both planar- and SPECT-derived parameters were not able to predict appropriate ICD therapy. These findings are line with a recent multicenter study including 135 CHF patients with ICD implantation for primary prevention.^20^

Previous large multicentre study and meta-analysis have suggested linear correlations between overall prognosis (i.e., progression CHF, cardiac death) and increased cardiac sympathetic activity, expect for AE and appropriated ICD therapy.^9^,^21^ However, there are some small studies suggesting an association between increase cardiac sympathetic activity and AE of appropriate ICD therapy. In a prospective study in 116 CHF patients, eligible for ICD implantation for both primary and secondary prevention of SCD, ^123^I-*m*IBG SPECT was shown to be an independent predictor of appropriate ICD therapy and cardiac death.^10^ The cumulative incidence of appropriate ICD therapy during 3-year follow-up was significantly higher when a relatively large ^123^I-*m*IBG SPECT defect (median summed score ≥26) was present. In a small study by Marshall et al^22^ including 27 CHF patients referred for ICD implantation with or without CRT, patients with AE, SCD had lower H/M ratios and higher ^123^I-*m*IBG SPECT defect score compared to those without an event.

A possible explanation for the difference in ^123^I-*m*IBG scintigraphy outcome in relation to appropriate ICD therapy between present study and previous studies^10^,^22^ could be related to differences in patient selection (i.e., ICD implantation for combined primary and secondary prevention), aetiology of CHF (i.e., ischemic vs. non-ischemic), differences in ICD settings, CRT, and number of patients with optimal medical therapy. More specifically the percentage of patients in the present study receiving ACE-inhibition (ACE-I) or angiotensin receptor blocker (ARB) was relatively low compared to other study cohorts.

The “bell-shaped” relation between ^123^I-*m*IBG and events is not new. For example, the results of the recent study by Verschure et al^20^ showed a “bell-shaped” curve for the early and late H/M ratio in relation to appropriated ICD therapy, suggesting that patients with intermediate late H/M ratio are more likely to have appropriate ICD therapy compared to patients with low and high late H/M ratios. Our findings are also in line with previous findings of Agostini et al showing that AE occurred in patients with an intermediate standardized late H/M ratio (1.46-2.17). In addition, Travin et al demonstrated similar results for ^123^I-*m*IBG SPECT defects in a population with 471 ischemic CHF patients, showing that, those with intermediate defects on ^123^I-*m*IBG SPECT summed score appeared to be at the highest risk for cardiac events.^23^ They concluded that the presumption of a monotonic increase in risk of an AE with increasing ^123^I-*m*IBG SPECT defects may not be correct. In line with these previous studies, the present study showed the same “bell-shaped” curve for late H/M ratio (Figure 1). For the SPECT-derived parameters similar curves were seen (data not shown). This underlines the notion that most likely there is no linear association between risk of an AE and ^123^I-*m*IBG myocardial uptake.

Although the exact pathophysiology of arrhythmias is still a matter of debate, it has been recognized that scar tissue and ischemia may serve as substrate for arrhythmias. Areas with slow conduction may facilitate the development of reentrant tachycardia.^24^ Especially the border zone of infarct tissue with viable myocardial tissue is predisposed to develop reentrant circuits. Sympathetic nerve fibers are more susceptible to ischemia than myocytes, thereby causing a disbalance between still viable but partly denervated and normal myocardium.^25^ This disbalance in myocardial sympathetic innervation may create a myocardial substrate particularly vulnerable to arrhythmia and arrhythmic death. The results of the present study with the “bell-shaped” curve for the late H/M ratio in relation to appropriated ICD therapy underline this hypothesis.

Recently the DANISH trail, reported that the ICD implantation for primary prevention of SCD in non-ischemic heart failure was not associated with improved survival compared with usual clinical care.^26^ However, SCD occurred less often in the ICD group compared to the control group [HR .50 (.31-.82), *P* = .005]. This suggests that some non-ischemic CHF patients remain at an increased risk for SCD. Future studies will be needed to confirm whether myocardial ^123^I-*m*IBG scintigraphy is helpful in specifically identifying non-ischemic CHF patients with an increased SCD risk.

^123^I-*m*IBG scintigraphy is a highly reproducible technique to assess cardiac sympathetic activity and has a small inter- and intra-observer variation.^27^ The analysis of ^123^I-*m*IBG SPECT can be challenging in patients with impaired myocardial ^123^I-*m*IBG uptake, i.e., discrimination between myocardium (low/no uptake) and lung (higher uptake) might be difficult. Although this difficulty might possibly interfere with the reading of the images, the present study showed a low variability in SPECT score between the 3 observers. This indicates that with visual scoring, observers are able to adequately overcome possible variation in a uniform way. In addition, the results of the present study are in line with previous studies showing a relatively small inter-observer variability.^27^,^28^

Despite the fact that ^123^I-*m*IBG scintigraphy is highly reproducible, there are many inter-institutional differences in image acquisition and post-processing. Therefore, international efforts have been made to harmonize and standardize cardiac ^123^I-*m*IBG scintigraphy.^14^ These recommendations include proposals for patient preparation, administered dose of ^123^I-*m*IBG activity (MBq), scanning parameters, and analysis of the acquired data to obtain the most used semi-quantitative parameters (i.e., early and late H/M ratio and ^123^I-*m*IBG washout (WO)). In addition, it has been suggested to correct for different collimator use and use standardized planar-derived parameters.^29^ This will further the clinical role of myocardial ^123^I-*m*IBG scintigraphy.

### Study Limitations

Although the included patients were treated with optimal medical therapy, the percentage of subject receiving ACE-inhibition/angiotensin receptor blocker was relatively low compared to other studies. This could have influenced the cardiac sympathetic activity in these patients and consequently the outcome of this study. Furthermore, the CHF aetiology of the enrolled patients was heterogeneous including both ischemic and non-ischemic HF. Therefore, additional studies are needed to establish the specific role of ^123^I-*m*IBG imaging in these specific subpopulations.

Although we did not perform myocardial perfusion imaging, this could have generated additional information on regional innervation/perfusion mismatch areas that might predispose to ventricular arrhythmias.^22^,^30^ Finally, although a ME collimator is recommended, in the present study a LEHR collimator was used. To overcome the difference in collimator use and to extrapolate these results to other institutions, standardized planar-derived parameters should be used.^29^

## Conclusion

In conclusion, SPECT ^123^I-*m*IBG scintigraphy-derived ESS was associated with AE in patients with ICD implantation for primary and secondary prevention. Although no linear association was found between ^123^I-*m*IBG scintigraphy-derived parameters and appropriate ICD therapy, there was a “bell-shaped” relation between ^123^I-*m*IBG scintigraphy-derived parameters and AE and appropriate ICD therapy. This seemingly counterintuitive finding implies that not those with the largest ^123^I-*m*IBG abnormalities tend to be at the highest risk for events but those with intermediate ^123^I-*m*IBG abnormalities.

## New Knowledge Gained

The present study showed a “bell-shaped” relation between cardiac sympathetic activity and AE and appropriated ICD therapy. These findings are in line with previous studies and underline the change in paradigm that not those with the largest ^123^I-*m*IBG abnormalities tend to be at the highest risk for events but those with intermediate ^123^I-*m*IBG abnormalities.

## Electronic supplementary material

Below is the link to the electronic supplementary material.

Supplementary material 1 (PPTX 1019 kb)

## Abbreviations

^123^I-*m*IBG: ^123^I-*meta*-iodobenzylguanidine
AE: Arrhythmic events
CHF: Chronic heart failure
DSS: Difference summed score
ESS: Early summed score
H/M ratio: Heart-to-mediastinum ratio
LSS: Late summed score
LVEF: Left ventricular ejection fraction
SCD: Sudden cardiac death
WO: Washout
