# Supplementary material for: Role of cardiac 123I-mIBG imaging in predicting arrhythmic events in stable chronic heart failure patients with an ICD
Source: J Nucl Cardiol. 2018 Mar 28;26(4):1188–96. doi: 10.1007/s12350-018-1258-z (PMC6660500; doi:10.1007/s12350-018-1258-z)
Supplement: Supplementary file 1 — Supplementary material 1 (PPTX 1019 kb) [file 12350_2018_1258_MOESM1_ESM.pptx]

## Slide 1
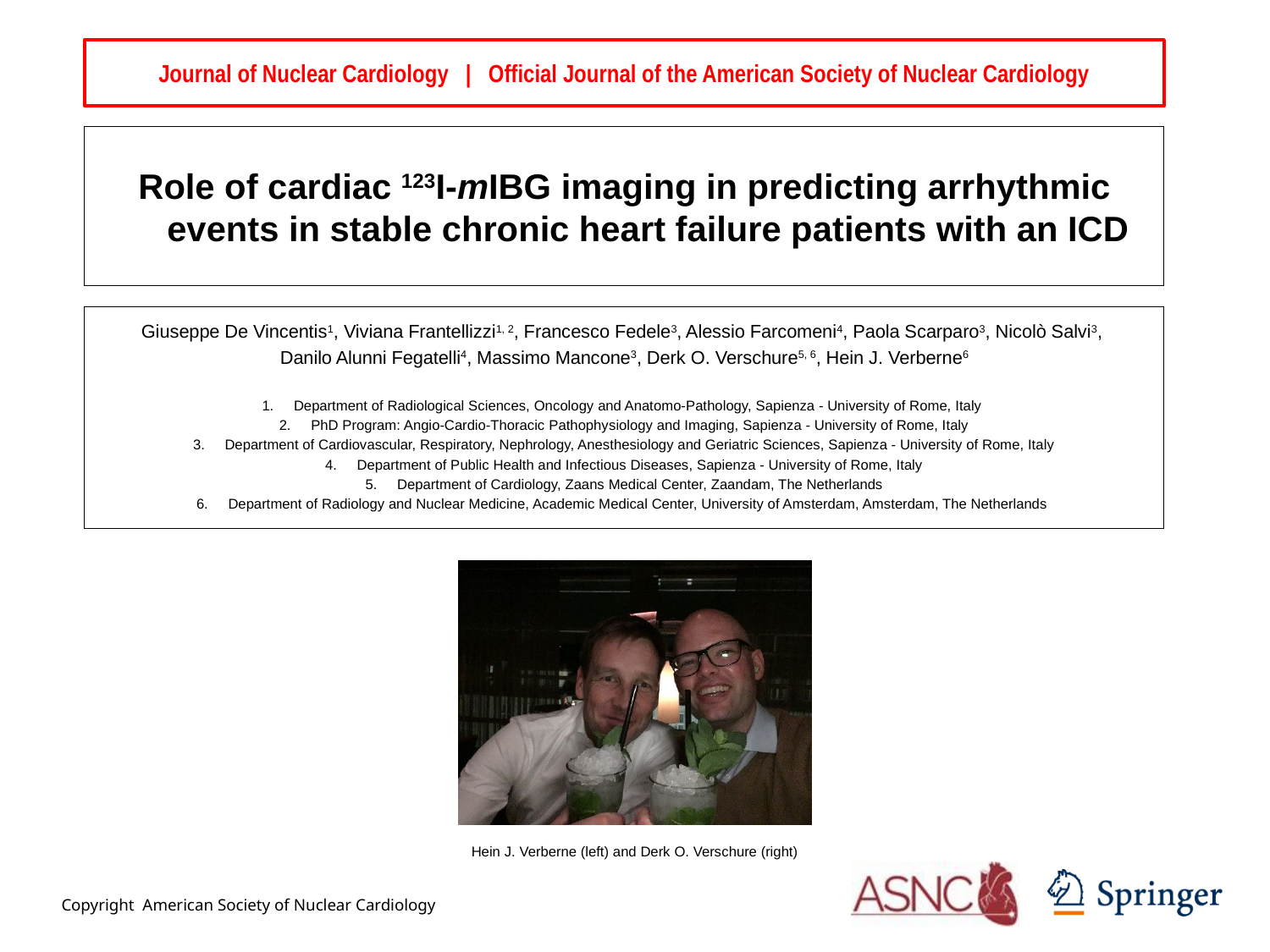

Journal of Nuclear Cardiology | Official Journal of the American Society of Nuclear Cardiology
# Role of cardiac 123I-mIBG imaging in predicting arrhythmic events in stable chronic heart failure patients with an ICD
Giuseppe De Vincentis1, Viviana Frantellizzi1, 2, Francesco Fedele3, Alessio Farcomeni4, Paola Scarparo3, Nicolò Salvi3,
Danilo Alunni Fegatelli4, Massimo Mancone3, Derk O. Verschure5, 6, Hein J. Verberne6
Department of Radiological Sciences, Oncology and Anatomo-Pathology, Sapienza - University of Rome, Italy
PhD Program: Angio-Cardio-Thoracic Pathophysiology and Imaging, Sapienza - University of Rome, Italy
Department of Cardiovascular, Respiratory, Nephrology, Anesthesiology and Geriatric Sciences, Sapienza - University of Rome, Italy
Department of Public Health and Infectious Diseases, Sapienza - University of Rome, Italy
Department of Cardiology, Zaans Medical Center, Zaandam, The Netherlands
Department of Radiology and Nuclear Medicine, Academic Medical Center, University of Amsterdam, Amsterdam, The Netherlands
Hein J. Verberne (left) and Derk O. Verschure (right)
Copyright American Society of Nuclear Cardiology

## Slide 2
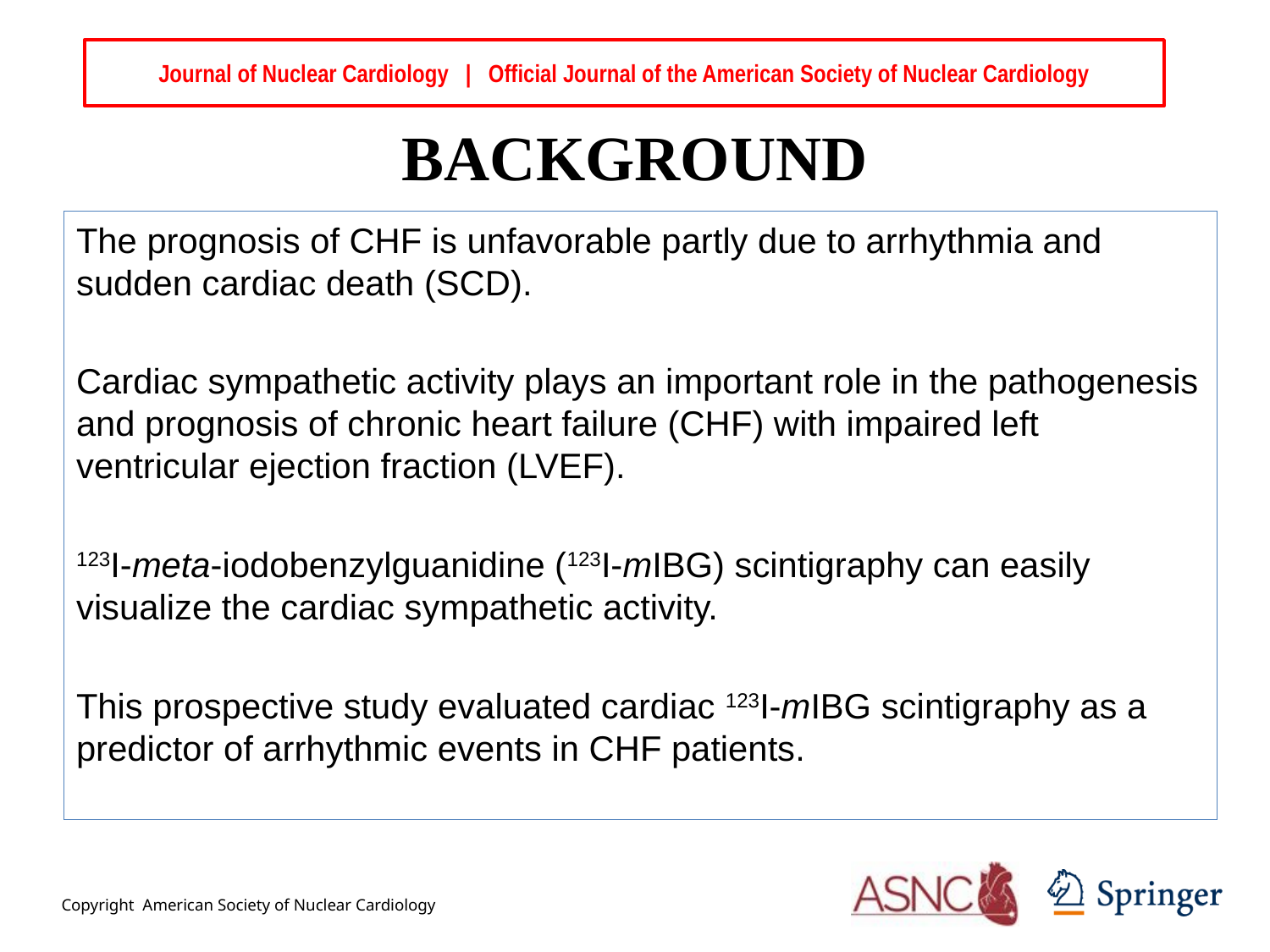

Journal of Nuclear Cardiology | Official Journal of the American Society of Nuclear Cardiology
# BACKGROUND
The prognosis of CHF is unfavorable partly due to arrhythmia and sudden cardiac death (SCD).
Cardiac sympathetic activity plays an important role in the pathogenesis and prognosis of chronic heart failure (CHF) with impaired left ventricular ejection fraction (LVEF).
123I-meta-iodobenzylguanidine (123I-mIBG) scintigraphy can easily visualize the cardiac sympathetic activity.
This prospective study evaluated cardiac 123I-mIBG scintigraphy as a predictor of arrhythmic events in CHF patients.
Copyright American Society of Nuclear Cardiology

## Slide 3
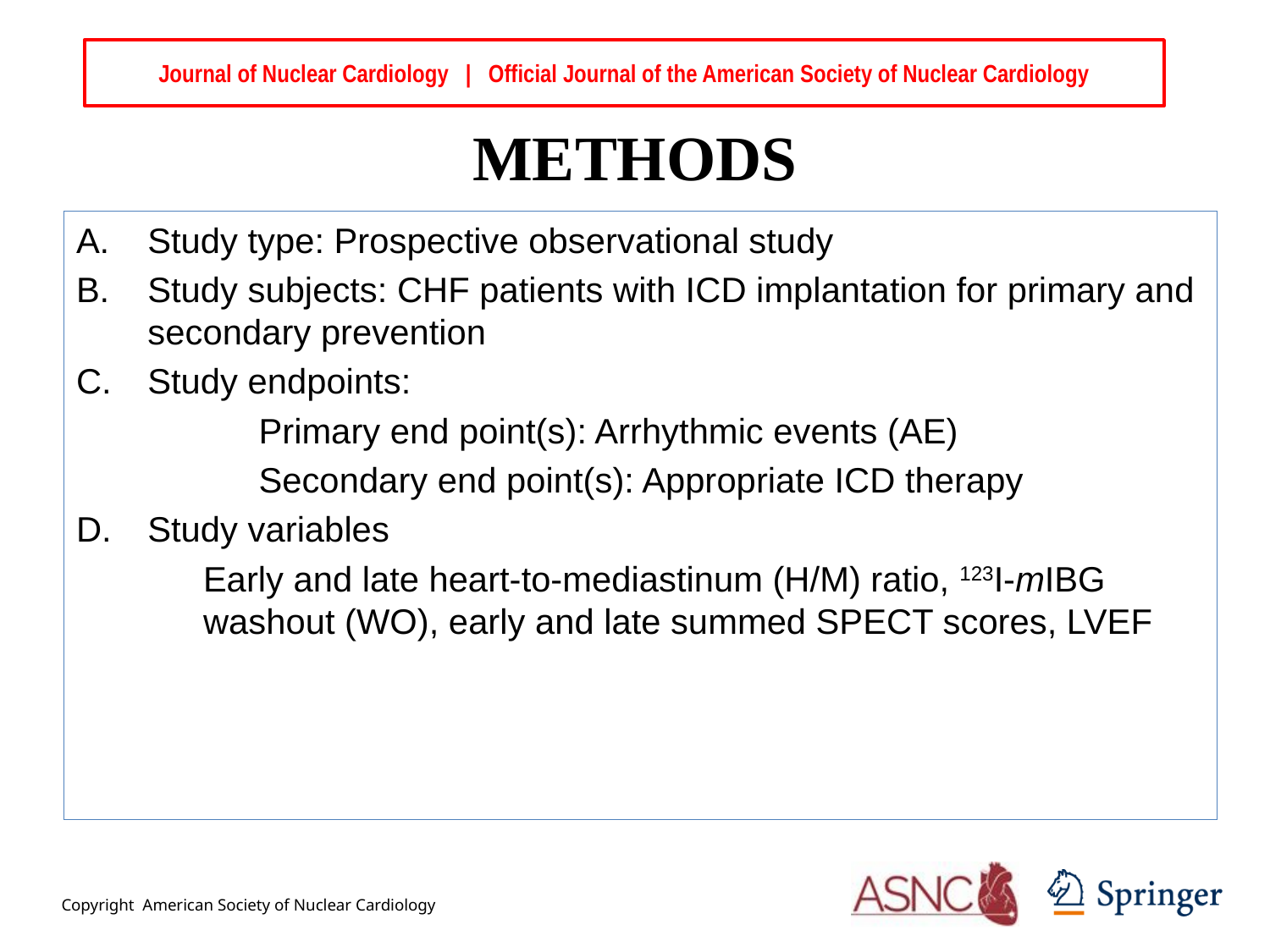

Journal of Nuclear Cardiology | Official Journal of the American Society of Nuclear Cardiology
# METHODS
Study type: Prospective observational study
Study subjects: CHF patients with ICD implantation for primary and secondary prevention
Study endpoints:
	Primary end point(s): Arrhythmic events (AE)
	Secondary end point(s): Appropriate ICD therapy
Study variables
	Early and late heart-to-mediastinum (H/M) ratio, 123I-mIBG 	washout (WO), early and late summed SPECT scores, LVEF
Copyright American Society of Nuclear Cardiology

## Slide 4
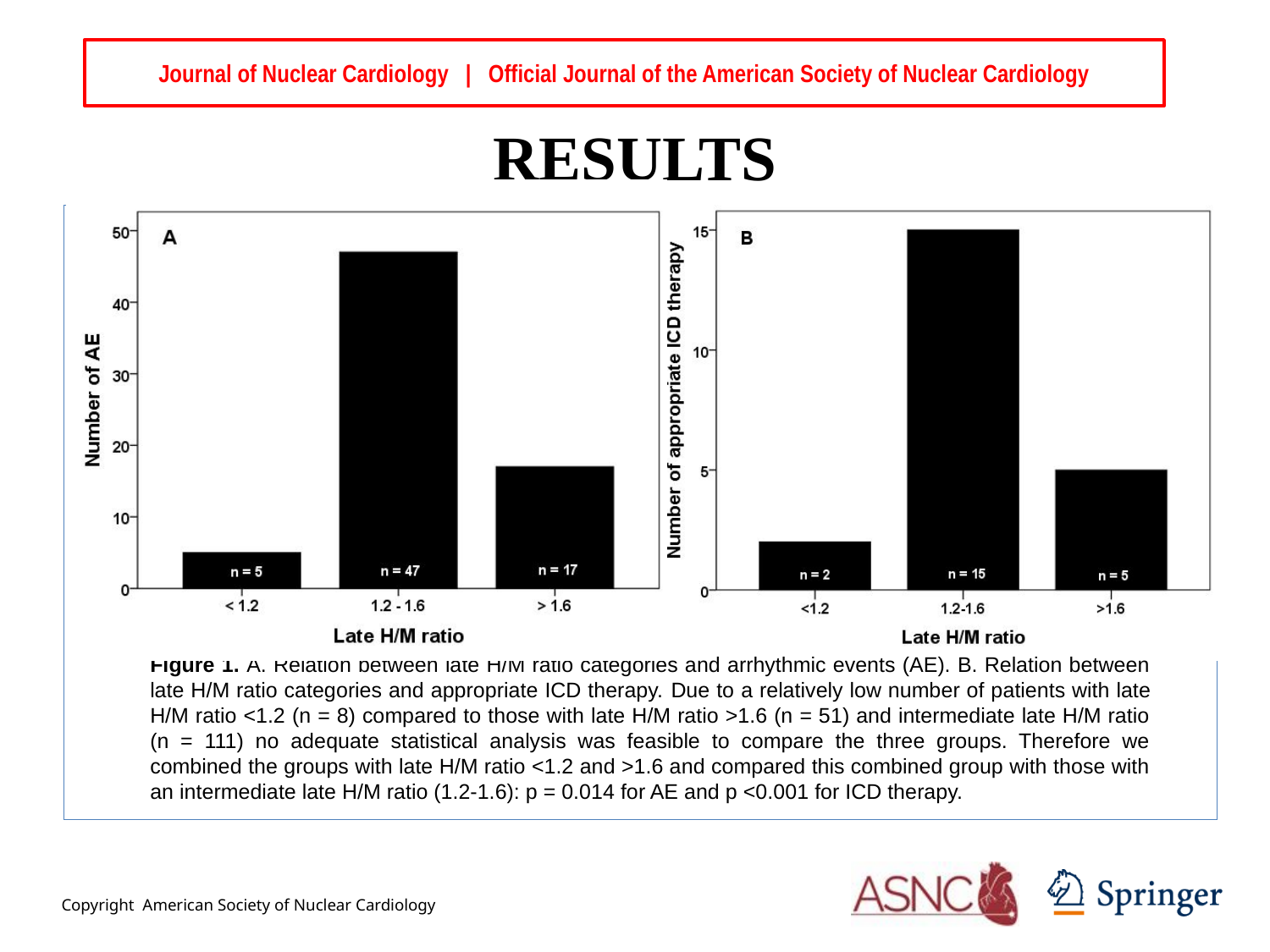

Journal of Nuclear Cardiology | Official Journal of the American Society of Nuclear Cardiology
# RESULTS
Insert a key table or a key figure
If figure, insert legend
Figure 1. A. Relation between late H/M ratio categories and arrhythmic events (AE). B. Relation between late H/M ratio categories and appropriate ICD therapy. Due to a relatively low number of patients with late H/M ratio <1.2 (n = 8) compared to those with late H/M ratio >1.6 (n = 51) and intermediate late H/M ratio (n = 111) no adequate statistical analysis was feasible to compare the three groups. Therefore we combined the groups with late H/M ratio <1.2 and >1.6 and compared this combined group with those with an intermediate late H/M ratio (1.2-1.6): p = 0.014 for AE and p <0.001 for ICD therapy.
Copyright American Society of Nuclear Cardiology

## Slide 5
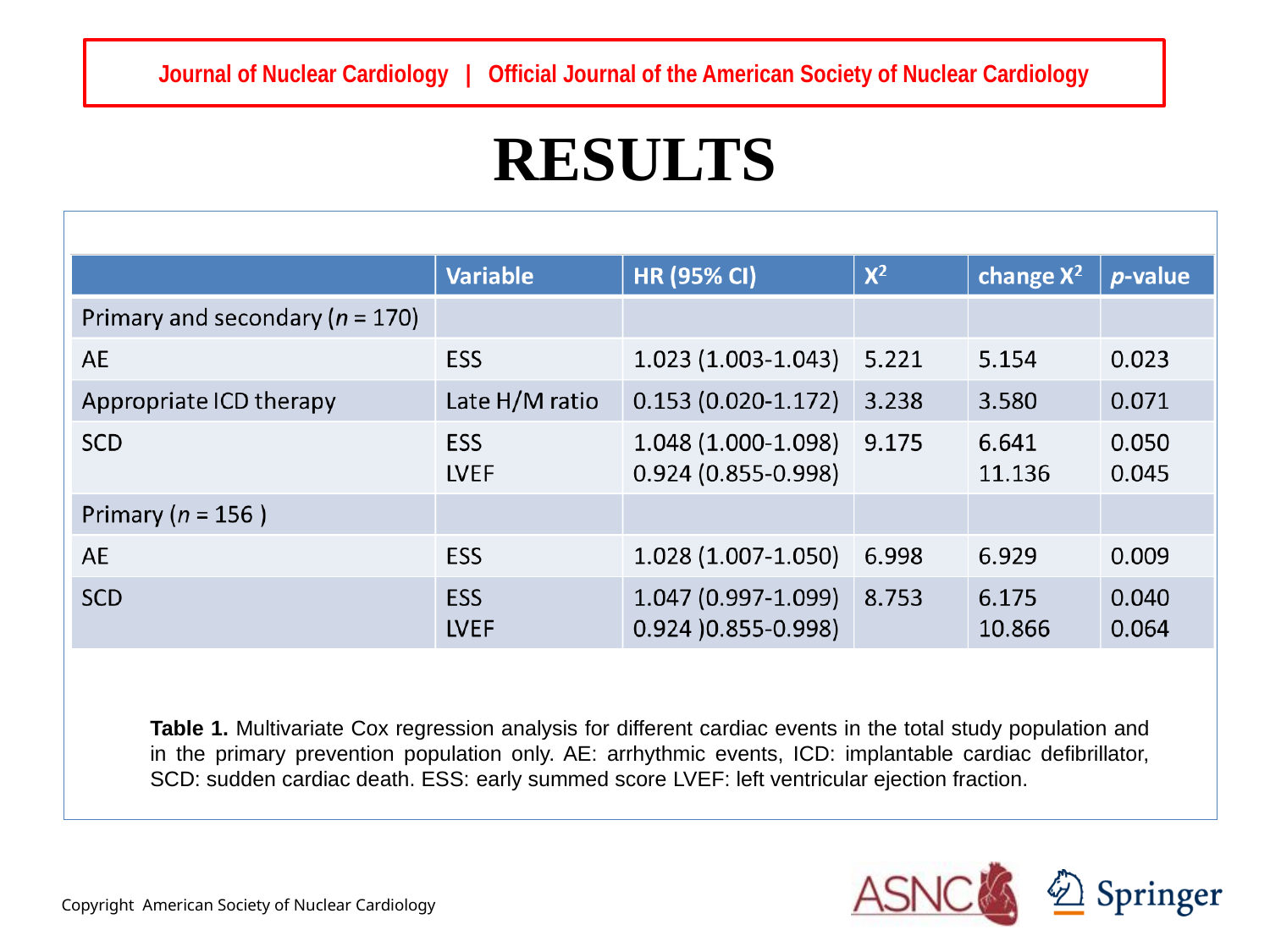

Journal of Nuclear Cardiology | Official Journal of the American Society of Nuclear Cardiology
# RESULTS
Insert a key table or a key figure
If figure, insert legend
Table 1. Multivariate Cox regression analysis for different cardiac events in the total study population and in the primary prevention population only. AE: arrhythmic events, ICD: implantable cardiac defibrillator, SCD: sudden cardiac death. ESS: early summed score LVEF: left ventricular ejection fraction.
Copyright American Society of Nuclear Cardiology

## Slide 6
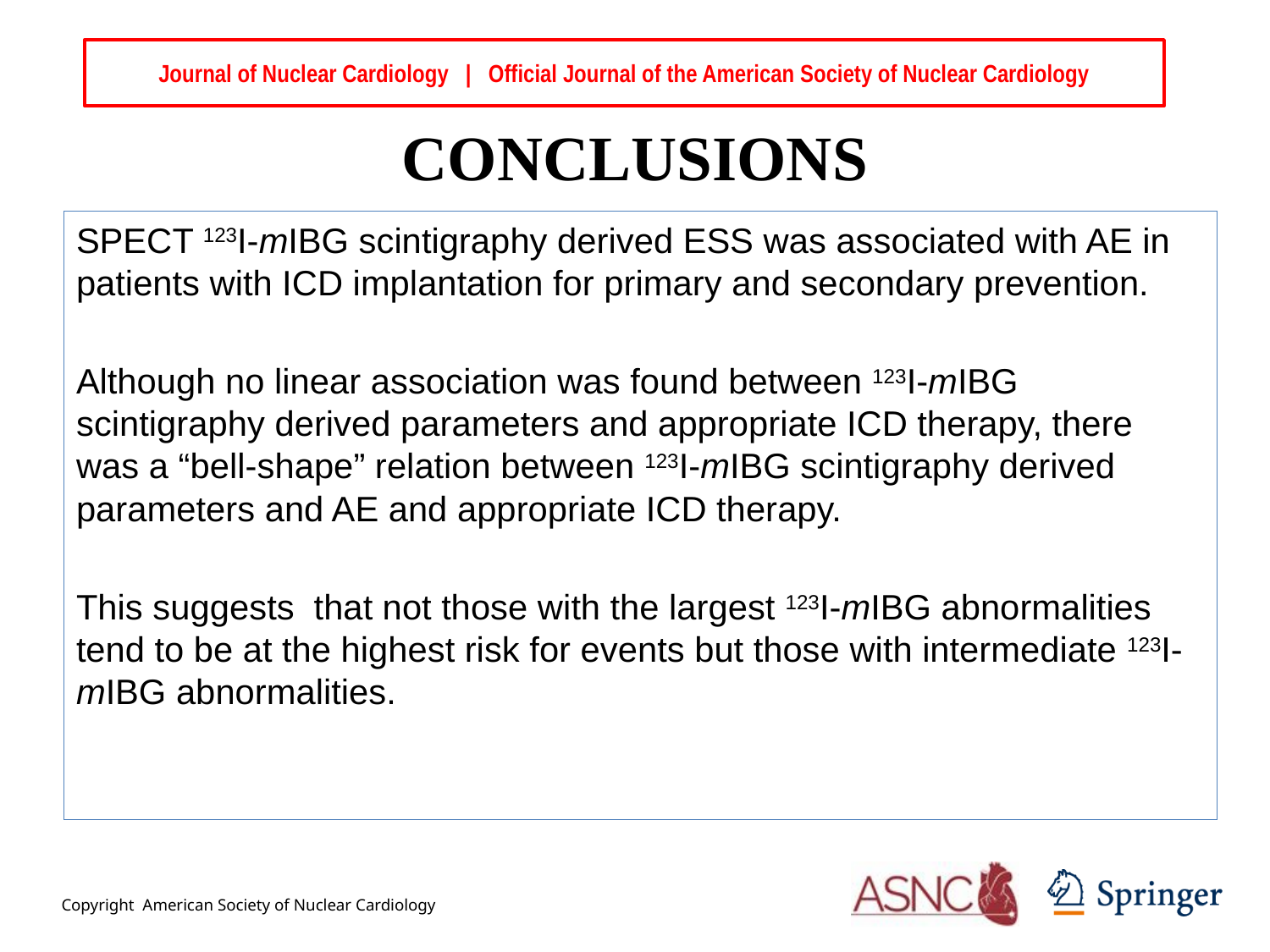

Journal of Nuclear Cardiology | Official Journal of the American Society of Nuclear Cardiology
# CONCLUSIONS
SPECT 123I-mIBG scintigraphy derived ESS was associated with AE in patients with ICD implantation for primary and secondary prevention.
Although no linear association was found between 123I-mIBG scintigraphy derived parameters and appropriate ICD therapy, there was a “bell-shape” relation between 123I-mIBG scintigraphy derived parameters and AE and appropriate ICD therapy.
This suggests that not those with the largest 123I-mIBG abnormalities tend to be at the highest risk for events but those with intermediate 123I-mIBG abnormalities.
Copyright American Society of Nuclear Cardiology
